# High Prevalence of Obesity, Hypertension, Hyperlipidemia, and Diabetes Mellitus in Japanese Outpatients with Schizophrenia: A Nationwide Survey

**DOI:** 10.1371/journal.pone.0166429

**Published:** 2016-11-17

**Authors:** Takuro Sugai, Yutaro Suzuki, Manabu Yamazaki, Kazutaka Shimoda, Takao Mori, Yuji Ozeki, Hiroshi Matsuda, Norio Sugawara, Norio Yasui-Furukori, Yoshitake Minami, Kurefu Okamoto, Toyoaki Sagae, Toshiyuki Someya

**Affiliations:** 1 Department of Psychiatry, Niigata University Graduate School of Medical and Dental Sciences, Niigata, Japan; 2 Japanese Society of Clinical Neuropsychopharmacology, Tokyo, Japan; 3 Japan Psychiatric Hospital Association, Tokyo, Japan; 4 Department of Psychiatry, Dokkyo Medical University School of Medicine, Mibu, Japan; 5 Department of Neuropsychiatry, Hirosaki University School of Medicine, Hirosaki, Japan; 6 Department of Health and Nutrition, Yamagata Prefectural Yonezawa University of Nutrition Sciences Faculty of Health and Nutrition, Yonezawa, Japan; University of Miami School of Medicine, UNITED STATES

## Abstract

**Background:**

Patients with schizophrenia have significantly shorter life expectancy than the general population, and a problem they commonly face is an unhealthy lifestyle, which can lead to obesity and metabolic syndrome. There is a very clear need to determine the prevalence of obesity, hypertension, hyperlipidemia, and diabetes mellitus which are components of metabolic syndrome in patients with schizophrenia, but there has been a paucity of large-scale studies examining this situation in Japan. The aim of our study was to address this need.

**Setting & Participants:**

We conducted a large-scale investigation of the prevalence of obesity, hypertension, hyperlipidemia, and diabetes mellitus using a questionnaire in 520 outpatient facilities and 247 inpatient facilities of the Japan Psychiatric Hospitals Association between January 2012 and July 2013. There were 7,655 outpatients and 15,461 inpatients with schizophrenia.

**Results:**

The outpatients had significantly higher prevalence of obesity, hypertension, hypertriglyceridemia, hyper-LDL cholesterolemia, and diabetes mellitus than the inpatients. The prevalence of hypo-HDL cholesterolemia was higher in inpatients than outpatients. Age-specific analysis showed the prevalence of obesity, hypertension, hypertriglyceridemia, hyper-LDL cholesterolemia, and diabetes mellitus among outpatients to be 2- to 3-fold higher than among inpatients. In individuals aged ≥60 years, the prevalence of obesity and DM among outpatients was about 3-fold higher than among inpatients.

**Conclusion:**

Japanese outpatients with schizophrenia were more likely to have physical risk such as obesity, hypertension, hyperlipidemia, and diabetes mellitus than inpatients. The physical risk to patients with schizophrenia may be affected by environmental parameters, such as type of care. The physical risk to Japanese patients with schizophrenia demands greater attention.

## Introduction

Patients with schizophrenia are at higher risk of mortality than individuals without the condition; patients with schizophrenia have a life expectancy approximately 20% shorter than the general population [1]. Patients with schizophrenia commonly have an unhealthy lifestyle, characterized by poor diet selection. Such patients are at an elevated risk of weight gain, leading to obesity and metabolic syndrome [2–4]. Some studies have reported that the prevalence of metabolic syndrome among patients with schizophrenia could range from approximately 20% to 40%. One population-based prevalence study found that patients with schizophrenia showed an elevated incidence of diabetes mellitus (DM), hypertension (HT), and hyperlipidaemia [5]. According to a study conducted in several institutions in Taiwan, the prevalence of hypertriglyceridemia (35.2%) and low levels of high-density lipoprotein (HDL) (42.6%) in patients with schizophrenia were higher than in the healthy group [6]. It has been reported that patients with acute-phase schizophrenia have poorer lipid profiles, such as lower HDL and higher low-density lipoprotein (LDL), which are related to the risk of developing cardiovascular disease and DM [7]. Central obesity, HT, hyperglycaemia, and dyslipidaemia are the core problems of metabolic syndrome that contribute to the high prevalence of cardiovascular disease [8]. Several studies have reported increased mortality with cardiovascular disease [9]. Japanese patients with schizophrenia may therefore have a higher mortality risk through obesity, HT, hyperlipidemia, and DM than the general population.

In Japan, most psychiatric care is entrusted to private psychiatric hospitals, the majority of which belong to the Japan Psychiatric Hospitals Association. In that country, 66.7% of all inpatients in psychiatric hospitals had been hospitalized for over 1 year at the time of the present investigation, and the mean duration of hospitalization of Japanese patients with schizophrenia is longer than with such patients in Europe and North America [10]. Differences in health-care systems between Japan and other countries could therefore affect the incidence of obesity, HT, hyperlipidemia, and DM. Despite the importance of examining the prevalence of these physical risk in patients with schizophrenia, few large-scale studies have investigated these problems in Japan. In the present study, we used a large questionnaire survey in a joint project to establish the prevalence of obesity, HT, hyperlipidemia, and DM in Japanese patients with schizophrenia.

## Methods

The survey was approved by the Ethics Committee of the Japan Psychiatric Hospitals Association. Written informed consent was obtained from all participants. The Japan Psychiatric Hospitals Association comprises 1217 facilities. In an investigation conducted by the Ministry of Health, Labour and Welfare in 2008, the number of Japanese patients with schizophrenia was found to be 795,000 [11], and the majority of them were treated by the Japan Psychiatric Hospitals Association. A joint project with the cooperation of the Japan Psychiatric Hospitals Association and the Japanese Society of Clinical Neuropsychopharmacology, with the aim of protecting patients with schizophrenia, was started in Japan in December 2012.

### Subjects

**Fig 1:**
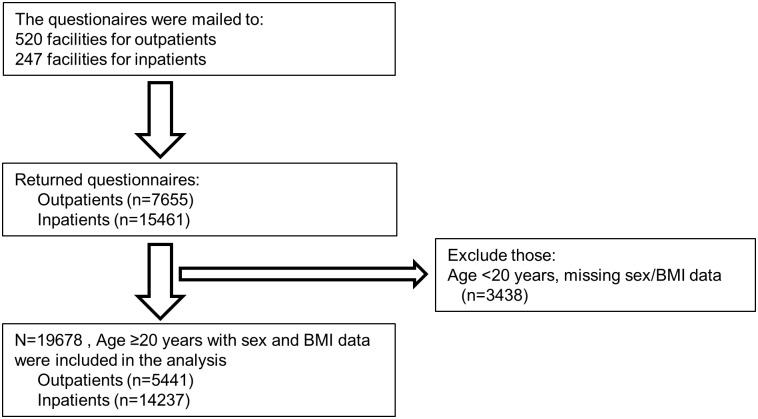
Flow diagram of participant inclusion and exclusion.

We conducted a questionnaire survey between January 2012 and July 2014. We obtained responses from 7655 outpatients and 15,461 inpatients in 520 facilities for outpatients and 247 facilities for inpatients belonging to the Japan Psychiatric Hospitals Association. All the patients were diagnosed with schizophrenia based on the Diagnostic and Statistical Manual of Mental Disorders, fourth edition, text revision, or the International Statistical Classification of Diseases and Related Health Problems, version 10. We excluded individuals aged under 20 years and those whose gender and body mass index (BMI) data were not assessed (n = 3,438). We analysed a final total of 19,678 individuals (5,441 outpatients, 14,237 inpatients; Fig 1).

### Measurements

After reviewing the relevant literature and guidelines, we compiled a brief questionnaire covering demographic data (age and gender), body height and weight, waist circumference (WC), blood pressure (BP), total cholesterol (TC), triglyceride (TG), LDL cholesterol, HDL cholesterol, and fasting plasma glucose (FPG) levels. BP was measured twice using a standard mercury sphygmomanometer while the individual was seated after at least a 5-minute rest. BMI was determined as the ratio of weight to height (kg/m^2^). Body height, weight, waist circumference and blood pressure were measured by skilled medical staff. Standardized health questionnaires were used to determine behaviour, including current smoking status. Medical staff also checked the data of TC, TG, LDL, HDL, and FPG by medical record within most recent three months. TC, TG, LDL, HDL, LDL/HDL ratio and FPG were also measured using standard analytical techniques.

### Definitions

In the present study, we investigated the prevalence of obesity, HT, hypertriglyceridemia, hyper-LDL cholesterolemia, hypo-HDL cholesterolemia and DM as physical risk of patients with schizophrenia. We applied the definition of obesity in the Asian-Pacific region (BMI ≥25 kg/m^2^) to categorize obesity [12]. DM was defined on the basis of FPG ≥126 mg/dl. Individuals were diagnosed as having HT if systolic pressure was ≥130 mmHg or diastolic BP ≥85 mmHg. Hypertriglyceridemia was defined as TG ≥150 mg/dl. Hyper-LDL cholesterolemia was defined as LDL ≥140 mg/dl. Hypo-HDL cholesterolemia was defined as HDL<40 mg/dl in males and <50 mg/dl in females.

### Statistical analysis

To compare the main demographic and clinical characteristics between the groups, we performed the unpaired Student’s *t* test to analyse continuous variables and the chi-square test to analyse categorical variables. A multivariate logistic regression analysis was performed to assess the effect of type of care, age and gender as a risk factor for obesity, HT, hypertriglyceridemia, hyper-LDL cholesterolemia, hypo-HDL cholesterolemia and DM. The threshold for significance was set at *p*<0.05. SPSS for Windows, version 19.0 (IBM Japan, Tokyo, Japan) was used for statistical calculations.

## Results

### Prevalence of obesity, HT, hypertriglyceridemia, hyper-LDL cholesterolemia, hypo-HDL cholesterolemia and DM and clinical characteristics

Tables 1 and 2 show the prevalence of obesity, HT, hypertriglyceridemia, hyper-LDL cholesterolemia, hypo-HDL cholesterolemia and DM and clinical characteristics for the outpatients, inpatients and Japanese general population. Compared with intpatients, outpatients were significantly higher BMI, WC, systolic BP, diastolic BP, LDL, LDL/HDL ratio, TC, TG, and FPG levels. The outpatients had significantly higher prevalence of obesity, HT, hypertriglyceridemia, hyper-LDL cholesterolemia, and DM than the inpatients. The inpatients had significantly lower prevalence of obesity, HT, hypertriglyceridemia, and hyper-LDL cholesterolemia than the general population. The prevalence of hypo-HDL cholesterolemia was higher in the inpatients than the outpatients and the general population. The inpatients were also significantly older and had lower BMI and WC than the outpatients. These results were independent of gender. There was a significant difference in antipsychotic therapy status between the outpatients and inpatients; total antipsychotics (chlorpromazine equivalents) administered were greater in the inpatients than in outpatients.

**Table 1:** Demographics and clinical characteristics between outpatients and inpatients.

	Outpatients	Inpatients	p value
	n = 5441	n = 14237	
Age (years)	52.2 ± 13.7	60.0 ± 12.9	<0.001^a^
Body mass index (kg/m^2^)	25.3 ± 4.6	22.3 ± 4.0	<0.001^a^
Waist circumference (cm)	87.6 ± 12.8	83.4 ± 11.5	<0.001^a^
Systolic blood pressure (mmHg)	127.2 ± 18.2	120.4 ± 17.2	<0.001^a^
Dyastolic blood pressure (mmHg)	78.2 ± 12.4	74.5 ± 12.1	<0.001^a^
HDL-cholesterol (mg/dl)	55.9 ± 19.0	55.0 ± 19.9	NS^a^
LDL-cholesterol (mg/dl)	117.6 ± 34.9	106.9 ± 44.8	<0.001^a^
LDL/HDL ratio	2.3 ± 1.0	2.1 ± 1.0	<0.001^a^
Total-cholesterol (mg/dl)	196.7 ± 38.4	177.7 ± 37.1	<0.001^a^
Triglycerides (mg/dl)	141.9 ± 99.4	101.0 ± 58.7	<0.001^a^
Fasting plasma glucose (mg/dl)	108.4 ± 40.3	92.6 ± 24.4	<0.001^a^
Na (mEq/l)	—	140.1 ± 4.4	—
K (mEq/l)	—	4.2 ± 0.5	—
Status of antipsychotic therapy			
No treated (%)	6.2	7.3	<0.001^b^
Antipsychotic monopharmacy (%)	49.4	40.3	
Antipsychotic polypharmacy (%)	44.4	52.4	
Ratio of SGA therapy (%)	73.3	75.8	<0.001^b^
Total CP equivalence (mg)	532.0 ± 472.6	691.1 ± 622.3	<0.001^a^
Smoking (%)	36.2	24.0	<0.001^b^

^a^ Data are analyzed using unpaired student's t test between outpatients and inpatient.

^b^ Data are analyzed using χ2 test between outpatients and inpatients.

Data are expressed as mean ± SD, CP, chlorpromazine; HbA1c, haemoglobin A1c; SGA, second-generation antipsychotic.

NS.; Not Significant, HDL; High density lipoprotein cholesterol,

LDL; Low density lipoprotein cholesterol, TC; Total cholesterol, TG; Triglyceride.

**Table 2:** The prevalence of obesity, HT, hypertriglyceridemia, hyper-LDL cholesterolemia, hypo-HDL cholesterolemia and DM among outpatients, inpatients and general population.

	Outpatients % (n)	Inpatients % (n)	General population^a^ % (n)
Obesity (%)	48.9^¶^ ^§^ (2659 / 5441)	23.1^#^ (3288 / 14237)	24.7 (1479 / 5985)
Hypertension (%)	30.5^¶^ ^§^ (1423 / 4667)	19.9^#^ (2734 / 13728)	27.2 (781 / 2871)
Hypertriglyceridemia (%)	33.3^¶^ (1281 / 3884)	14.5^#^ (1451 / 10024)	29.8 (894 / 2997)
Hyper-LDL-cholesterolemia (%)	23.9^¶^ (530 / 2214)	14.8^#^ (954 / 6467)	22.2 (649 / 2924)
Hypo-HDL-cholesterolemia (%)	14.7^¶^ (346 / 2349)	18.1^#^ (1058 / 5861)	15.3 (460 / 2997)
Diabetes mellitus (%)	16.8^¶^ ^§^ (551 / 3287)	7.1 (321 / 4529)	10.9 (384 / 3508)

^¶^Significant difference was observed between outpatients and inpatients.

^§^Significant difference was observed between outpatients and general population.

^#^Significant difference was observed between inpatients and general population.

Hypertriglyceridemia was defined as TG ≥150 mg/dl.

Hyper-LDL-cholesterolemia was defined as LDL ≥140 mg/dl.

Hypo-HDL-cholesterolemia was defined as HDL<40 mg/dl in male and <50 mg/dl in female.

Diabetes mellitus was defined as FPG ≥ 126 mg/dl.

^a^ Data of general population are quoted from national survey by Ministry of Health and Welfare in 2011.

### Age-specific prevalence of obesity, hypo-HDL choresterolemia and DM

Tables 3–5 show the prevalence of obesity, hypo-HDL choresterolemia and DM in the outpatients, inpatients, and Japanese general population according to age [13]. The overall prevalence of obesity, HT, hypertriglyceridemia, hyper-LDL cholesterolemia, and DM among the outpatients was higher than among the inpatients. In particular, among individuals aged ≥60 years, the prevalence of obesity and DM among the outpatients was about 3-fold higher than among the inpatients. With older individuals, the prevalence of obesity and DM among the inpatients was lower than among the general population. Although the prevalence of obesity in male inpatients was lower than in the general population aged ≥50 years, female inpatients showed a higher prevalence of obesity than the general population at a younger age. Among individuals aged ≤59 years, the prevalence of hypo-HDL cholesterolemia among the inpatients was higher than among the general population, although there were no difference in the prevalence of hypo-HDL cholesterolemia between the outpatients and the general population. The prevalence of HT, hypertriglyceridemia and hyper-LDL cholesterolemia among the outpatients were also higher than among the inpatients.

**Table 3:** Age-specific Prevalence of obesity, hypo-HDL cholesterolemia and DM among outpatients, inpatients and general population (Total).

	Age (years)	Total		
		Outpatients % (n)	Inpatients % (n)	General population^a^ % (n)
Obesity	20–29	42.1^¶^ ^§^(104 / 247)	27.8^#^ (76 / 273)	15.0 (69 / 459)
	30–39	47.8^¶^ ^§^ (402 / 841)	32.1^#^ (268 / 835)	22.2 (177 / 797)
	40–49	54.6^¶^ ^§^ (672 / 1230)	34.0^#^ (621 / 1827)	27.2 (222 / 816)
	50–59	54.7^¶^ ^§^ (706 / 1290)	25.9 (787 / 3038)	27.9 (256 / 916)
	60–69	43.2^¶^ ^§^ (555 / 1284)	20.3^#^ (1006 / 4954)	27.6 (334 / 1210)
	≥70	40.1^¶^ ^§^ (220 / 549)	16.0^#^ (530 / 3310)	26.3 (398 / 1514)
Hypo-HDL cholesterolemia	20–29	10.7 (9 / 84)	19.0^#^ (22 / 116)	8.5 (17 / 200)
	30–39	14.9 (49 / 329)	17.9^#^ (66 / 368)	12.1 (54 / 448)
	40–49	16.1 (81 / 503)	19.0^#^ (146 / 767)	12.7 (52 / 411)
	50–59	14.8^¶^ ^§^ (86 / 581)	19.3^#^ (242 / 1252)	10.0 (48 / 482)
	60–69	15.0 (89 / 593)	17.3 (347 / 2011)	15.6 (103 / 659)
	≥70	12.4^§^ (32 / 259)	17.4^#^ (235 / 1347)	23.3 (186 / 797)
Diabetes Mellitus	20–29	7.7^§^ (11 / 142)	4.5 (3 / 66)	0.5 (1 /199)
	30–39	9.3^§^ (47 / 503)	5.5^#^ (14 / 255)	1.8 (8 / 449)
	40–49	14.2^¶^ ^§^ (107 / 753)	5.6 (34 / 606)	3.5 (15 / 427)
	50–59	18.4^¶^ ^§^ (146 / 795)	6.3 (60 / 953)	7.6 (41 / 537)
	60–69	22.3^¶^ ^§^ (168 / 754)	8.2^#^ (131 / 1597)	16.8 (141 / 841)
	≥70	21.2^¶^ (72 / 340)	7.5^#^ (79 / 1052)	16.9 (178 / 1055)

^¶^Significant difference was observed between outpatients and inpatients;

^§^Significant difference was observed between outpatients and general population;

^#^Significant difference was observed between inpatients and general population.

Hypertriglyceridemia was defined as TG ≥150 mg/dl; Hyper-LDL-cholesterolemia was defined as LDL ≥140 mg/dl; Hypo-HDL-cholesterolemia was defined as HDL<40 mg/dl in male and <50 mg/dl in female; Diabetes mellitus was defined as FPG ≥ 126 mg/dl.

^a^ Data of general population are quoted from national survey by Ministry of Health and Welfare in 2011.

**Table 4:** Age-specific Prevalence of obesity, hypo-HDL cholesterolemia and DM among outpatients, inpatients and general population (Male).

	Age (years)	Male		
		Outpatients % (n)	Inpatients % (n)	General population^a^ % (n)
Obesity	20–29	42.7^¶^ ^§^ (53 / 124)	21.9 (35 / 160)	21.2 (43 / 203)
	30–39	49.2^¶^ ^§^ (239 / 486)	30.6 (152 / 496)	32.9 (122 / 371)
	40–49	55.6^¶^ ^§^ (429 / 771)	30.9 (325 / 1053)	34.8 (128 / 368)
	50–59	54.5^¶^ ^§^ (421 / 772)	22.7^#^ (397 / 1748)	33.4 (144 / 431)
	60–69	43.3^¶^ ^§^ (325 / 750)	17.9^#^ (472 / 2633)	31.5 (171 / 542)
	≥70	38.9^¶^ ^§^ (95 / 244)	13.5^#^ (194 / 1437)	26.2 (175 / 668)
Hypo-HDL cholesterolemia	20–29	20.0 (7 / 35)	19.7 (14 / 71)	11.0 (8 / 73)
	30–39	22.0 (44 / 200)	25.2^#^ (54 / 214)	13.9 (25 / 179)
	40–49	21.1 (63 / 298)	25.1^#^ (112 / 446)	14.0 (20 / 143)
	50–59	22.1^§^ (77 / 348)	26.5^#^ (190 / 716)	11.7 (22 / 188)
	60–69	21.0^§^ (73 / 347)	24.5^#^ (267 / 1090)	13.3 (38 / 285)
	≥70	17.2 (20 / 116)	24.3 (138 / 568)	21.3 (79 / 371)
Diabetes Mellitus	20–29	8.1^§^ (5 / 62)	7.3 (3 / 41)	0.0 (0 / 73)
	30–39	10.7^¶^ ^§^ (31 / 290)	4.4 (7 / 160)	2.2 (4 / 180)
	40–49	14.7^¶^ ^§^ (66 / 449)	4.7 (16 / 338)	6.7 (10 / 150)
	50–59	20.8^¶^ ^§^ (99 / 476)	7.4 (41 / 552)	10.8 (23 / 213)
	60–69	25.6^¶^ (113 / 441)	7.8^#^ (66 / 845)	22.7 (80 / 353)
	≥70	18.4^¶^ (29 / 158)	9.1^#^ (40 / 440)	23.1 (109 / 473)

^¶^Significant difference was observed between outpatients and inpatients;

^§^Significant difference was observed between outpatients and general population;

^#^Significant difference was observed between inpatients and general population.

Hypertriglyceridemia was defined as TG ≥150 mg/dl; Hyper-LDL-cholesterolemia was defined as LDL ≥140 mg/dl; Hypo-HDL-cholesterolemia was defined as HDL<40 mg/dl in male and <50 mg/dl in female; Diabetes mellitus was defined as FPG ≥ 126 mg/dl.

^a^ Data of general population are quoted from national survey by Ministry of Health and Welfare in 2011.

**Table 5:** Age-specific Prevalence of obesity, hypo-HDL cholesterolemia and DM among outpatients, inpatients and general population (Female).

	Age (years)	Female		
		Outpatients % (n)	Inpatients % (n)	General population^a^ % (n)
Obesity	20–29	41.5^§^ (52 / 123)	36.3^#^ (41 / 113)	10.2 (26 / 256)
	30–39	45.9^¶^ ^§^ (163 / 355)	34.2^#^ (116 / 339)	12.9 (55 / 426)
	40–49	52.9^¶^ ^§^ (243 / 459)	38.2^#^ (296 / 774)	21.0 (94 / 448)
	50–59	55.0^¶^ ^§^ (285 / 518)	30.2^#^ (390 / 1290)	23.1 (112 / 485)
	60–69	43.1^¶^ ^§^ (230 / 534)	23.0 (534 /2321)	24.4 (163 / 668)
	≥70	41.0^¶^ ^§^ (125 / 305)	17.9^#^ (336 / 1873)	26.4 (223 / 846)
Hypo-HDL cholesterolemia	20–29	4.1 (2 / 49)	17.8 (8 / 45)	7.1 (9 / 127)
	30–39	3.9^§^ (5 / 129)	7.8 (12 / 154)	10.8 (29 / 269)
	40–49	8.8 (18 / 205)	10.6 (34 / 321)	11.9 (32 / 268)
	50–59	3.9^¶^ ^§^ (9 / 233)	9.7 (52 / 536)	8.8 (26 / 294)
	60–69	6.5^§^ (16 / 246)	8.7^#^ (80 / 921)	17.4 (65 / 374)
	≥70	8.4^§^ (12 / 143)	12.5^#^ (97 / 779)	25.1 (107 / 426)
Diabetes Mellitus	20–29	7.5^¶^ ^§^ (6 / 80)	0.0 (0 / 100)	0.8 (1 / 126)
	30–39	7.5^§^ (16 / 213)	7.4^#^ (7 / 95)	1.5 (4 / 269)
	40–49	13.5^¶^ ^§^ (41 / 304)	6.7^#^ (18 / 268)	1.8 (5 / 277)
	50–59	14.7^¶^ ^§^ (47 / 319)	4.7 (19 / 401)	5.6 (18 / 324)
	60–69	17.6^¶^ (55 / 313)	8.6^#^ (65 / 752)	12.5 (61 / 488)
	≥70	23.6^¶^ ^§^ (43 / 182)	6.4^#^ (39 / 612)	11.8 (69 / 583)

^¶^Significant difference was observed between outpatients and inpatients;

^§^Significant difference was observed between outpatients and general population;

^#^Significant difference was observed between inpatients and general population.

Hypertriglyceridemia was defined as TG ≥150 mg/dl; Hyper-LDL-cholesterolemia was defined as LDL ≥140 mg/dl; Hypo-HDL-cholesterolemia was defined as HDL<40 mg/dl in male and <50 mg/dl in female; Diabetes mellitus was defined as FPG ≥ 126 mg/dl.

^a^ Data of general population are quoted from national survey by Ministry of Health and Welfare in 2011.

### The effect of type of care on the odds ratio for obesity, HT, hypertriglyceridemia, hyper-LDL cholesterolemia, hypo-HDL cholesterolemia and DM

To assess the independent effect of type of care for schizophrenia on the odds ratio for obesity, HT, hypertriglyceridemia, hyper-LDL cholesterolemia, hypo-HDL cholesterolemia and DM, a logistic regression analysis was performed (Table 6). Except for hypo-HDL cholesterolemia, the odds ratios of having obesity, HT, hypertriglyceridemia, hyper-LDL cholesterolemia, and DM were significantly higher for outpatients when analysed with age and gender as covariates.

**Table 6:** Logistic regression analysis of obesity, HT, hypertriglyceridemia, hyper-LDL cholesterolemia, hypo-HDL cholesterolemia and DM in subjects.

Independent variables	Covariate	P value	OR (95%CI)
Obesity	Age	< 0.001	0.982 (0.979 to 0.984)
	Male	< 0.001	0.830 (0.778 to 0.884)
	Outpatients	< 0.001	2.823 (2.637 to 3.022)
Hypertension	Age	< 0.001	1.013 (1.010 to 1.016)
	Male	< 0.001	1.310 (1.220 to 1.407)
	Outpatients	< 0.001	1.911 (1.767 to 2.066)
Hypertriglyceridemia	Age	< 0.001	0.988 (0.985 to 0.991)
	Male	< 0.001	1.330 (1.218 to 1.451)
	Outpatients	< 0.001	2.656 (2.428 to 2.905)
Hyper-LDL cholesterolemia	Age	NS	1.002 (0.998 to 1.006)
	Male	< 0.001	0.734 (0.655 to 0.822)
	Outpatients	< 0.001	1.879 (1.661 to 2.127)
Hypo-HDL cholesterolemia	Age	NS	1.001 (0.997 to 1.006)
	Male	< 0.001	3.142 (2.756 to 3.583)
	Outpatients	< 0.001	0.749 (0.653 to 0.859)
Diabetes Mellitus	Age	< 0.001	1.019 (1.014 to 1.025)
	Male	< 0.001	1.235 (1.068 to 1.429)
	Outpatients	< 0.001	3.045 (2.613 to 3.548)

Hypertriglyceridemia was defined as TG ≥150 mg/dl; Hyper-LDL-cholesterolemia was defined as LDL ≥140 mg/dl; Hypo-HDL-cholesterolemia was defined as HDL<40 mg/dl in male and <50 mg/dl in female; Diabetes mellitus was defined as FPG ≥ 126 mg/dl; NS: not significant

## Discussion

In the present nationwide survey, we found the prevalence of obesity, HT, hypertriglyceridemia, hyper-LDL cholesterolemia, and DM in Japanese outpatients with schizophrenia to be higher than in inpatients with schizophrenia. With older individuals, the prevalence of HT, hypertriglyceridemia, hyper-LDL cholesterolemia, and DM among inpatients was lower than in the general population. Only the prevalence of hypo-HDL cholesterolemia among the inpatients was higher than among the outpatients and the general population. To the best of our knowledge, the present study is the largest survey to clarify the difference in the prevalence of obesity, HT, hypertriglyceridemia, hyper-LDL cholesterolemia, hypo-HDL cholesterolemia and DM between Japanese outpatients and inpatients with schizophrenia.

### Difference in the physical risk between the outpatients and inpatients

Many studies have found schizophrenia to be associated with obesity, and DM. Some reports have found DM among patients with schizophrenia to be 2-fold higher than in the general population [5, 14]. The increased incidence of obesity, and DM among patients with schizophrenia is associated with lifestyle factors [15, 16]. However, there have been few previous studies that compared the prevalence of obesity, HT, hypertriglyceridemia, hyper-LDL cholesterolemia, and DM in outpatients with those in inpatients. Although our study design could not control the potential confounding factors such as lifestyle factors, genetic factors, and medication effects, we identified the prevalence of obesity, HT, hypertriglyceridemia, hyper-LDL cholesterolemia, and DM between Japanese outpatients and inpatients in this large-scale survey. A major finding in the present study was that the outpatients had a significantly higher prevalence of obesity, HT, hypertriglyceridemia, hyper-LDL cholesterolemia, and DM than the inpatients.

Other studies have also established a higher prevalence of HT in outpatients than in inpatients [17, 18]. Schizophrenia-related HT has been associated with weight gain and metabolic syndrome through patients’ sedentary lifestyles [19]. The present study also identified a higher risk of developing hyperlipidaemia in outpatients with schizophrenia. Obesity in individuals with an unhealthy lifestyle of reduced physical activity and poor diet is more common in outpatients with schizophrenia than inpatients, and this could also be related to hyperlipidaemia. In the general population, it has been said that hyperlipidaemia may play a primary role in the development of atherosclerotic vascular disease and subsequent death due to the disease [20]. Previous study suggest that LDL /HDL ratio are risk indicator for atherosclerosis with greater predictive value than each lipid parameter used independently [21]. Another study suggests that patients with schizophrenia had higher LDL / HDL ratio than healthy general population. In the present study, there were significant difference in LDL/HDL ratio between the outpatients and inpatients [22]. This finding also suggest that outpatients with schizophrenia may have higher risk for development of dyslipidaemia and atherosclerotic vascular disease. However, there may be an overall underestimation in the findings related to this issue owing to uneven recognition and management of cholesterol in patients with schizophrenia [23].

### The mental health system in Japan

We were unable to establish why Japanese outpatients with schizophrenia had a higher prevalence of obesity, HT, hypertriglyceridemia, hyper-LDL cholesterolemia, and DM. One possible reason about these differences between outpatients and inpatients may be that long-term hospitalization has a protective effect on the physical health of patients with schizophrenia. The system for dealing with patients with schizophrenia in Japan is hospital-based, and it has the largest number of psychiatric beds per person in the world. Long-term hospitalization is a severe problem and leads to greater medical costs. However, it may affect the lower prevalence of obesity, HT, hypertriglyceridemia, hyper-LDL cholesterolemia, and DM in Japanese inpatients with schizophrenia compared with outpatients because the inpatients receive constant lifestyle management.

### Age and gender differences between the outpatients and inpatients

We also conducted an age-specific analysis of the prevalence of obesity, HT, hypertriglyceridemia, hyper-LDL cholesterolemia, and DM and found that among individuals aged ≥60 years, the prevalence of obesity and DM in outpatients was about 3-fold higher than in inpatients. Older age are strongly related to DM, HT, and hyperlipidaemia [24]. However, age-specific analyses of schizophrenia have found a higher incidence of these metabolic co-morbidities among younger patients [25]. It has been reported that the adjusted risk of DM in patients with schizophrenia was greater in females than in males for all age groups, the greatest risk in females being for those aged 20–29 years [5]. Our age-specific analysis also demonstrated that the prevalence of HT and DM was elevated in older age groups. Our findings reflect the greater vulnerability of older patients in developing HT and DM. In comparison with the general population, younger patients with schizophrenia (20–29 years) showed a greater likelihood of having hyperlipidaemia. Hyperlipidaemia may be common among young adults with schizophrenia owing to their higher prevalence of obesity, unhealthy dietary intake, and unhealthy lifestyles [26]. Additionally, there may be a greater likelihood of younger people being unaware of their hyperlipidaemia, and under-diagnosis may be more problematic for that age group [27]. Psychotic symptoms usually occur during adolescence or in young adulthood and then present a chronic pattern, which negatively affects the individual’s cognition, social and physical activity levels, and self-care. It is also necessary to note that metabolic disturbances may be independent of increased body weight and can be an early biochemical change after initiating antipsychotic use [28]. It is therefore important to make regular checks of the blood lipid profile for early detection and consequent treatment in patients with schizophrenia.

Gender differences associated with schizophrenia and the risk of hyperlipidaemia, HT, and DM deserve attention. Some studies have found female patients with schizophrenia to be at higher risk for metabolic syndrome [29, 30]. However, we found that the incidence of DM was slightly greater among males than among females. Because our findings are from a cross-sectional study and limited by some unavailable data, further investigations are needed to examine gender-specific issues in patients with schizophrenia.

In the present study, contrary to our expectations, only the prevalence of hypo-HDL cholesterolemia among the inpatients was higher than among the outpatients and the general population. It was reported that the most likely improvement in the lipid profile induced by physical activity is an increase in HDL cholesterol [31]. In hospital, where physical activity is relatively confined, it may be difficult to increase the value of HDL.

### Limitation

This study has limitations similar to those of other cross-sectional surveys of patients with schizophrenia and metabolic syndrome. Because we were unable to obtain information about the duration of illness and medication, we did not consider the influence of antipsychotics.

### Conclusion

We conducted a large questionnaire survey and observed that Japanese outpatients with schizophrenia were more likely to have physical risk such as obesity, HT, hypertriglyceridemia, hyper-LDL cholesterolemia, and DM than inpatients. The average length of hospitalization in Japan is the longest in the world, and there has been a gradual ageing of inpatients in Japan. This aspect of Japan’s mental health system may be related to the difference in physical risk between outpatients and inpatients with schizophrenia.
